# The experiences of attending an international veteran sporting competition and preparatory camp for Team Ukraine during the Russo-Ukrainian conflict

**DOI:** 10.3389/fspor.2024.1395672

**Published:** 2024-12-23

**Authors:** Claire L. Castle, Nikki Heinze, Renata S. M. Gomes

**Affiliations:** ^1^ Social and Welfare, BRAVO VICTOR, London, United Kingdom; ^2^ School of Music, Faculty of Arts, Humanities and Cultures, University of Leeds, Leeds, United Kingdom; ^3^ BRAVO VICTOR, London, United Kingdom; ^4^ Faculty of Health and Life Sciences, Northumbria University, Newcastle upon Tyne, United Kingdom

**Keywords:** sports, disability, veterans, military, conflict, sports competition, training, well-being

## Abstract

**Introduction:**

This article offers unique insight into Team Ukraine's experiences of attending an international sporting event for veterans living with disability and injured active-duty personnel (the United States' Department of Defense Warrior Games, “The Games”) and a 5-week preparatory camp in the United Kingdom (UK).

**Methods:**

A survey gathered qualitative data at three time points: during the second and final week of training camp, and the in the two weeks immediately following participation at The Games. Forty-four out of 55 members of Team Ukraine (including veterans, active-duty personnel, support staff, and family members) provided responses in Survey 1, 20 in Survey 2, and 18 in Survey 3. These responses were thematically analysed to explore experiences and motivations for participation.

**Results and discussion:**

Five main themes were identified: Rest and recuperation; The ongoing conflict as a source of motivation; The sporting experience; Veterans in sport; and Relationships. Whilst physically demanding, the camp was an opportunity for respite and emotional processing, and participants reflected on social benefits and improvements to physical health and sporting abilities. Perhaps of greatest significance was the role of the ongoing conflict in their motivations to attend The Games. The conflict brought participants together in their collective goals of representing Ukraine on an international stage, raising awareness of the war, and demonstrating strength through sporting success. Findings highlight the challenges experienced and overcome by Team Ukraine and provides insight into the role that competitive sporting events may play during times of conflict as a means of demonstrating national strength and pride, and meeting individuals' health and well-being needs.

## Introduction

In February 2022, Ukraine was invaded in a major escalation of the Russo-Ukrainian Conflict. In 2022, the United States Department of Defense invited, for the first time, Ukrainian veterans and injured active-duty military personnel to compete alongside American and Canadian service teams in the Warrior Games (“The Games”) in Florida. Sixty-one Ukrainian competitors and support staff attended The Games. This included a small number of family members of deceased personnel, and veterans (who returned to military duties during the conflict) and active-duty personnel permitted military leave to take part. Fifty-five of these individuals attended a 5-week preparatory training camp hosted by Blind Veterans UK in the United Kingdom (UK), which consisted of an intensive training schedule, psychotherapy sessions, and recreational trips. Expanding on current knowledge of sports, exercise, and competition during conflict, the current article seeks to explore Team Ukraine's experiences of preparing for, and taking part in, The Games. To date, this is the first exploration of a sports team's experiences of an international competition while their nation is at war.

### Sports participation amongst military personnel and veterans

There is growing evidence of the role of sports and exercise in meeting the physical, social, psychological, and rehabilitative needs of military personnel and veterans (1). Participation in structured exercise has been associated with physical benefits such as weight loss and increased fitness (2) and, in older veterans, increased mobility, strength, and aerobic capacity (3, 4). The psychological impacts of sports and exercise on veterans have also been documented, including its role in motivating individuals (2), the reduction of post-traumatic stress symptoms through outdoor and nature-based activities (5–8), increases in emotional capacities facilitated through social bonding, a sense of freedom, and a redefining or recapturing of identity (9). For veterans with disability, improvements in depression, anxiety, social functioning, and positive and negative affect have also been noted (10), along with positive associations between sport, exercise, and recreation (SER) and self-esteem and self-reported quality of life (11).

Research has also considered the impact of sporting competitions such as the US-based Warrior Games, National Veterans Wheelchair Games, National Veterans Summer Sports Clinic, and National Disabled Veterans Winter Sports Clinic, and the international multi-sport Invictus Games, on the lives and rehabilitative experiences of veterans (12–14). A systematic review of the benefits of sports and physical activity, including sporting competitions, for veterans, describes multiple benefits to subjective (e.g., active coping, positive affective experience), psychological (e.g., increased determination, focus on ability, improved self-concept), and social well-being (1). Literature documents the role of sporting events in providing veterans with disability with opportunities to increase sports-related skills and knowledge, acceptance of disability, and social interactions, and to improve physical and mental health (12, 13). Research relating to the Invictus Games suggests that competitive sports contribute positively to rehabilitative processes through several mechanisms, including motivation, and its targeting of the physical, psychological and social impacts of injury; participation might provide individuals with a greater understanding of their body and capabilities, a new purpose and focus in life, and a renewed military camaraderie (2). However, competitors may also be impacted negatively by a perceived lack of goals following the competition, “post-games blues” (13) (p. 3557), and stress before and during participation (13, 15).

### Sports participation during times of conflict

Despite the inclusion of military and veteran groups in research relating to SER, the role of sports and sporting events in the lives of veterans and military personnel during times of conflict remain underexplored. Such periods will inevitably hinder both individuals’ engagement in SER and a nation's involvement in international sporting events. Yet, sports may play an important role in the lives of those living through, and recovering from, traumatic events. There is evidence of the role of outdoor SER in meeting both the physical and psychological needs of individuals impacted by humanitarian crises, including the COVID-19 pandemic (16, 17) and natural disasters (18–20). There has been some consideration of sports participation during conflict, although this research has tended to focus on the experiences of children, young people, and refugees, and its role in post-conflict recovery (21, 22).

Beyond individual experience, sports may also have a wider role to play during times of conflict. There is acknowledgement of interactions between modern sport and domestic and international politics, with large sports events such as the Olympics and the World Cup bringing countries together, with the goal of closer and more cordial relations (23). Sporting events may become politicized in the context of war, contributing to both national expressions of identity and culture (24), and peace-making, through the promotion of government relations and international understanding (23). Feizabadi, Delgado and Pormennati (23) note that athletes have “a significant role to play in condemning military wars in the international arena” (p. 528). Given evidence of the negative psychophysiological experiences arising during conflict for both civilians and military groups (25, 26), the psychological challenges and benefits associated with competitive sports (27, 28), and the potential value of SER to individuals impacted by international conflict, the current article considers the question, “What role does competitive sport have to play in the lives of military personnel, veterans, and their support networks during an ongoing conflict?”.

This article is particularly timely, given the ongoing work being undertaken by The Ministry of Veterans Affairs of Ukraine, with support from the NATO Trust Fund for Medical Rehabilitation, on its National Strategy for physical and sports rehabilitation of war veterans and their families, approved in 2021 (29, 30). This ongoing work has, so far, seen the development of a mobile application which helps veterans and personnel access free sports rehabilitation (31) and the delivery of a rehabilitation programme for Ukrainian veterans suffering from post-traumatic stress disorder (PTSD) as a result of the Russian invasion (32).

## Materials and methods

The study consisted of three online surveys run with members of Team Ukraine, which included active personnel and veterans who had returned to active duties during the Russo-Ukraine conflict, and a small number of family members of personnel killed during military service. The research design was guided by the practical restraints imposed on data collection due to the unique context in which this work was undertaken. Survey methods were chosen as most appropriate for several reasons. First, participants’ time was limited due to the busy nature of their daily schedule during their time in the UK. Surveys enabled data collection to be carried out at a time that suited participants, for example, in-between training sessions or in the evening, with minimum disruption to their day. Second, knowledge of Team Ukraine's arrival in the UK was shared in a restricted circle only 14 days prior to their arrival for security reasons, with confirmation of dates and location given to the research team at this time. This limited the time available to develop the current study and plan delivery. Surveys removed the need to recruit Ukrainian-speaking research team members to run qualitative data collection, which contributed to ease of data collection during participants’ busy training and recreational schedule.

Surveys were delivered using the online survey platform Phonic. Survey 1 (S1) was carried out at the start of the training camp (in week 2), Survey 2 (S2) at the end of the camp (week 5), and Survey 3 (S3) at the end of The Games (during the 2 weeks following the end of The Games). S1 and S2 explored experiences of preparing for The Games, whilst S3 explored participants’ experiences at The Games. All three surveys included free text open-response questions, for which participants were able to type or audio record their responses. Whilst the data associated with one single individual's response to each open-ended question, and thus subsequent analyses, may lack the nuances and richness usually associated with qualitative research, open-ended response questions are common in survey research as a way of capturing the direct experiences of respondents, including perceptions and concerns (33, 34). These may be particularly useful during exploratory studies and phases (35–37). S1 and S2 also collected demographic data, including age, disability, gender, and role in the team, details of previous sporting experience, and quantitative data which explored indicators of health and well-being during the training camp. Quantitative findings relating to S1 and S2 data are reported elsewhere (14). Responses to the open-ended questions are the focus of the current analysis. Respondents in all three surveys were asked about their role in the team, to provide context to their open-ended responses (see Table 1). As in the article outlining quantitative findings (14), the current study was run as part of a service evaluation for the training camp and host institution and so did not require approval from an ethics panel; this was confirmed by the Chair of the Medical Sciences Interdivisional Research Ethics Committee at the University of Oxford.

**Table 1:** Participant's roles within team Ukraine.

		S1	S2	S3
		*n* = 44	*n* = 20	*n* = 19
Age	*M*	38.93	38.95	/
	*SD*	9.79	9.54	/
	Range	19–64	25–56	/
Gender	Female	14 (31.1%)	6 (30%)	/
	Male	30 (66.7%)	14 (70%)	/
Role in team	Athlete (active-duty personnel)	13.6% (6)	20.0% (4)	21.1% (4)
	Athlete (veteran)	22.7% (10)	20.0% (4)	21.1% (4)
	Athlete (veteran who re-joined active duty)	22.7% (10)	20.0% (4)	21.1% (4)
	Coach	13.6% (6)	5.0% (1)	15.8% (3)
	Family	9.1% (4)	15.0% (3)	10.5% (2)
	Support	18.2% (8)	20.0% (4)	10.5% (2)

### Survey questions

Open-ended questions in S1 explored motivations, concerns and challenges associated with taking part in The Games, and participants’ goals for the training camp. In S2, participants were asked to reflect on their training and/or time in the UK, motivational factors, and what they were most looking forward to during The Games. Finally, S3 asked participants about things they had learned, significant or memorable moments, and impacts of their involvement in The Games on their health and psychological state. The questions sought to cover both positive and negative aspects of experiences, aiming to identify factors identified in the literature as impacted by sporting participation for veterans with disability, such as increased sports-related skills and knowledge, social experiences, and physical and mental well-being (12, 13). All questions were translated into Ukrainian by a professional translator and checked by a member of Team Ukraine staff for accuracy. Open-ended questions from each of the surveys are provided in the Appendix. Questions were not validated, but were checked for sense-making and language by a member of Team Ukraine support staff.

As the first study to consider the experiences of individuals involved in an international sporting event during an ongoing international conflict, open-ended questions were broad and did not ask about specific motivations, goals, benefits or challenges, but rather allowed participants to elaborate on those aspects of the experience they felt were most important. This was also important given the inclusion of different groups within the sample (e.g., military veterans, serving personnel, spouses/family members, and support staff). In S3, participants were asked to reflect on any skills they felt they had developed, and any health and well-being benefits of participation. Due to the sensitive nature of asking about the ongoing conflict in Ukraine, particularly given the loss of friends and family members within the team, questions did not ask directly about experiences of war. It was hoped that, should such experiences be relevant, participants would raise these in their responses to questions about motivations for participation and experiences of representing Team Ukraine.

### Participants and recruitment

Participation was voluntary. All 55 training camp attendees were invited to participate by Team Ukraine management, who notified the team of the study. Participants could access the online survey using a link or QR code on printed flyers shared onsite, and via a message sent on the team's private communication app by team management. A total of 44 camp attendees provided qualitative responses in S1, 20 in S2, and 18 in S3. The high attrition between S1 and S2 may reflect the intensity of the training camp, increased tiredness, and greater focus on the upcoming competition towards the end of the camp, with a subsequent decreased interest in the survey. Based on demographic data gathered in S1 and S2, respondents were aged 25–56 years (*M* = 38.38 years, *SD* = 9.67) and six respondents were female (33%). Participant characteristics for each survey round are presented in Table 1.

### Procedure

Prior to taking part in S1, participants were required to provide informed consent. The opening page of the survey provided information about its aims, what was involved in participation, and how data would be used. This was followed by a series of consent statements, to which respondents had to agree before they could proceed to the survey. Participants reconfirmed their consent in the same way before completing S2 and S3. S1 was run the second week of Team Ukraine's time in the UK (22nd–26th July), S2 during their final days in the UK (14th–18th August), and S3 was completed after participation in The Games (27th August–6th September). Paper and English versions were available for S1 and S2. All participants completed S1 and S2 online in Ukrainian, thus S3 was delivered online and in Ukrainian only. Responses were translated from Ukrainian into English by a professional translation agency who are compliant with General Data Protection Regulation (GDPR) requirements.

### Analysis

**Figure 1:**
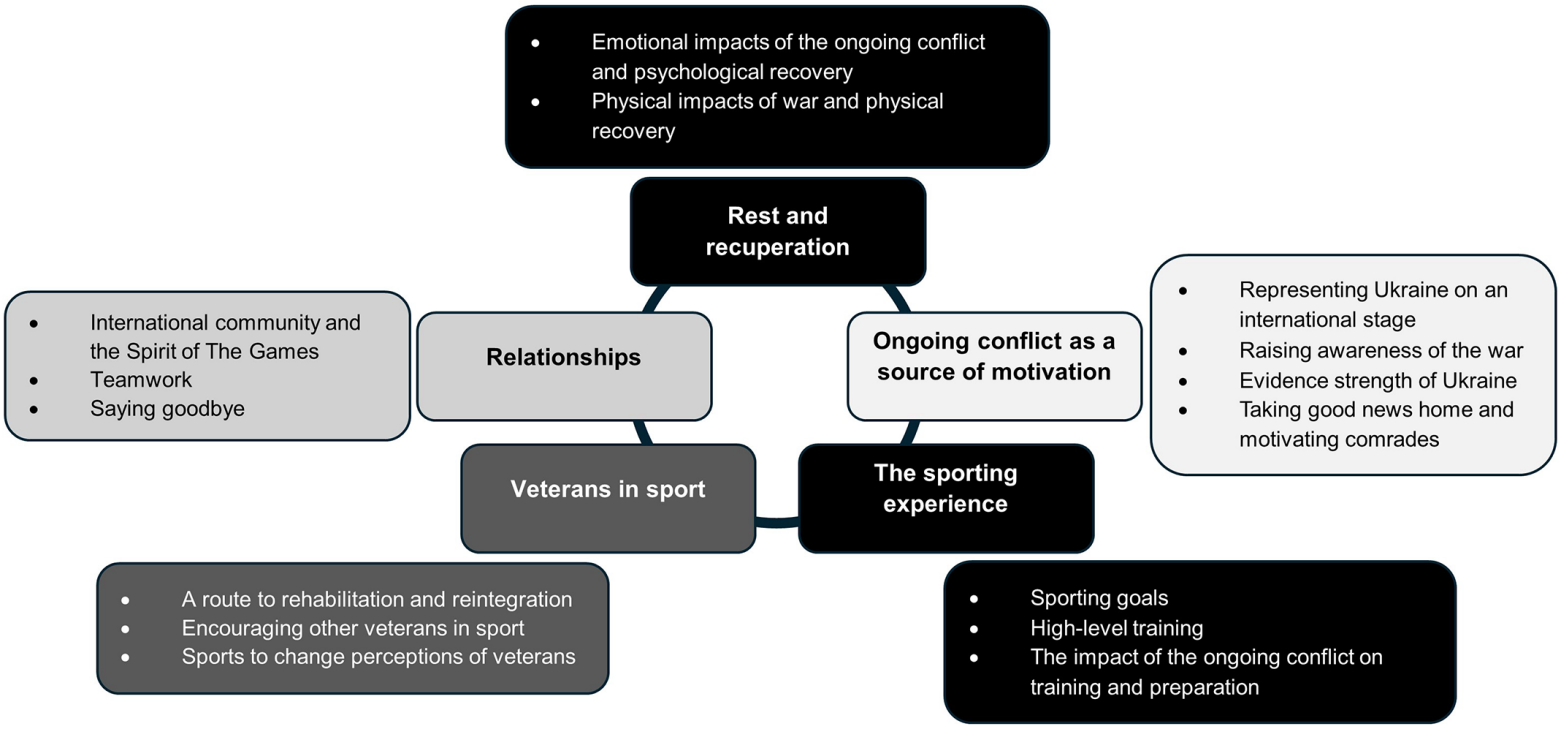
Thematic map.

Thematic analysis (TA) was used to identify and report patterns across the dataset (38). TA is not attached to a particular paradigmatic orientation and can be used across both positivist/quantitative and qualitative paradigms (38). The practical and theoretical flexibility of TA was valuable in the current study, given the pragmatic approach to data collection required to overcome the logistical limitations on project set-up and delivery. TA was undertaken in line with the six phase process described by Terry et al. (39), reflecting Braun and Clarke's original six-step process (38): (1) familiarisation (reading and re-reading); (2) generation of initial codes; (3) construction of preliminary themes (re-focusing analysis at a broader level, sorting codes into potential themes, and collating coded data extracts within the identified themes); (4) reviewing of themes (including combining themes or removing poorly supported themes); (5) defining and naming themes; and (6) report writing to communicate the analyst's story of the data. Analysis was inductive (data driven), which is particularly useful when exploring new areas of research (40). NVivo software was used to organise and collate themes and extracts. Whilst survey methods limit potential exploratory insight into the lived experiences of participants and the ability to uphold a truly “reflexive” approach (41), open-ended response data from the current project are still viewed as qualitatively valuable. Five main themes were identified, as seen in Figure 1: Rest and recuperation; The ongoing conflict as a source of motivation; The sporting experience; Veterans in sport; Relationships.

## Results

### Rest and recuperation

The backdrop of the ongoing conflict in Ukraine played an important part in many participants’ responses, reflected in comments regarding the impact of the war on both their physical health and emotional well-being. Research demonstrates the negative psychological impact of the conflict in Ukraine on both military and displaced civilian populations, including high levels of symptoms of stress, depression, isolation, anxiety, and intrusive memories (42). Indeed, in the current sample, there were family members attending in the place of personnel killed during the conflict; as comments below demonstrate, this was a highly traumatic experience with implications on their psychological state during the duration of the training camp and The Games.

### Physical impacts of war and physical recovery

Given the impact of the war on health and well-being, participants acknowledged the holistic benefits that the training camp offered for physical and mental rest, recuperation, and recovery from the war. Participation in the war had impacted on the physical health of many participants, including physical exhaustion and injury, the latter of greatest relevance to those involved in active military duties. As early as S1, participants acknowledged the time afforded to individuals at the training camp for recovery of physical strength, and the rehabilitative value of sporting participation.

First of all, competition, well, not competition, but sport for me is more like rehabilitation (Active-duty personnel, S1)

An opportunity to rest, gain strength, even sleep. At first, I didn't want to go at all, as I am a volunteer and help our army, and my help is important, since even sending one tourniquet to the front line can save someone’s life. But now I understand that this trip saved me, gave me the opportunity to recover, gain strength and feel how Britain supports us (Support staff, S1)

The impact is extremely positive. I regained my physical form, which had been somewhat lost since I had returned from the front line, being pretty exhausted from being in the war zone. Here I had the opportunity to recover, I actually regained my athletic form, powerfully rebooted myself and my mental health is now at the highest level (Support staff, S3)

With this sense of physical renewal came reflections on the implications of participant’s time in the UK on their future involvement in the ongoing conflict. Commenting in S3, one active member of personnel commented on the importance of the camp and The Games in improving physical fitness, given the ongoing hostilities in Ukraine and involvement of members of the team in the conflict, “physical fitness is one of the most important components of good military form”. Another noted that, “My health improved significantly and I was able to return to military service”. Thus, whilst the training camp was targeted at preparing participants for the upcoming Games, the camp and The Games were also considered an important means of allowing participants to both physically recover from, and prepare for, their role in the ongoing conflict. Existing literature has evidenced similar benefits of competitive sporting participation on the physical health and fitness of veterans, including increased physical functioning and mobility skills (2, 13). As participants in the current study highlighted, such benefits may be of particular importance to those recently involved in, or soon to return to, active military duties.

### Emotional impacts of the ongoing conflict and psychological recovery

Undoubtedly, the war had also taken an emotional toll of many members of Team Ukraine. The camp was considered a welcome psychological break from the conflict, as well as a time for emotional healing, particularly for those involved in active duty. The training camp provided a distraction from the ongoing conflict, with one veteran athlete reflecting in S3 on, “New experiences to distract me from problems”. Integral to this healing process was the nature of the camp as a safe environment away from the experiences of war.

There are no worries, just rest a little. After five months of war, everyone was a little tired. And constant uncertainty about the future scares everyone. And here you can work a little on [sport redacted], restore yourself (Veteran, S1)

First of all, we could be in the safest possible environment, where there are no explosions or rockets. It makes us feel more stable and safer psychologically (Veteran, S2)

There were also those managing experiences with death and grief during their stay in the UK. For the wife of a fallen soldier, who had taken his place on Team Ukraine, the training camp was an important opportunity to process her grief.

While I am on a trip with the team, I have time to work on myself, grieve and cry, go through the death of my husband together with his friends who help and support me… I am hoping to start a new life. I will have fond memories of my husband. I will work through the pain and start over (Wife of fallen soldier, S1)

Later in S2, this participant reflected on the challenging nature of this grief process, but also the benefits of spending time with those who knew her husband and were also managing this loss. Alongside professional psychological support, this factor appeared to play an important part in allowing this individual to work through negative emotions and leave the camp feeling less distressed.

These were psychological difficulties, related to memories of my deceased husband. I met the members of the team who are, who were his friends, brothers. And they supported me, and I had sessions with psychologists, and now I feel a little bit better (Wife of fallen soldier, S2)

Dooley et al. (43) suggest that the coming together of survivors of military losses in a positive environment enables military survivors to connect with each other, find a sense of belonging, learn they are not alone, and benefit from peer-based emotional support. The current study suggests that such an environment might be successfully fostered through sporting participation, and may be of particular importance to military personnel, families, and the wider civilian community, who experience loss during conflict.

Notably, even those for whom benefits relating to psychological recuperation were not evident in early comments (S1), these benefits were acknowledged later at S2 and S3. As one member of support staff commented in S1, their focus was to build motivation amongst competing members of the team, but at S2, they observed that for themselves, the camp had been a chance “to catch my breath, relax a little mentally and gather myself up physically”.

### A break from military duties vs. guilt

Despite the psychological benefits of time away from the war, feelings of guilt for leaving military duties, comrades, and loved ones, were not uncommon. For one participant, their biggest concern relating to participating in the camp at S1 had been, “That my family is left without protection for this period” (Veteran). Military service is a unique career that often requires frequent and prolonged absences from family due to deployments, temporary duties or relocation, which can lead to feelings of guilt (44). As with military duties, training and competing took members of Team Ukraine away from their family, at a time where their family's safety was at greatest risk. Similarly, there was guilt amongst those who had left their military duties at this critical time, with concern for those left behind.

It was very difficult to leave my country. Since I can't rest and train knowing my friends and warriors are being killed every day (Support staff, S1)

My moral and psychological state, worries about my sworn brothers and sisters, remorse for leaving my unit (Serving veteran, S2)

Research commonly refers to “survivor guilt”, experienced by those exposed to, or witness to, death, but coming to no harm themselves (45). “Survivor guilt” may manifest due to one of several beliefs held by the surviving individual: that they themselves should have died as well as, or instead of, another person; or that they are responsible in some way for the death of another (45). In the current study, guilt appeared to be tied to potential for the latter, with concerns regarding the danger that comrades faced in their absence and their inability to contribute as others did whilst away. Clearly, time spent away from military duties led to such feelings for some, and the decision to attend the training camp and Games was not an easy one to make. There was a sense of some participants having to weigh-up the pros and cons of attendance, typified by one participant actively involved in military duties in Ukraine.

This year it was very difficult to make a choice and go to the training and the competition itself, as there is a lot of work at home, namely, to protect the state of Ukraine… But after talking with my compatriots, I heard their support and request to convey the true information about the war in our country to everyone whom we will meet (Active-duty personnel, S1)

### The ongoing conflict as a source of motivation

Whilst the war had created physical and emotional challenges for participants, the ongoing conflict was also integral to participants’ decisions to participate in The Games. For many, the war provided a source of motivation. Firstly, it instigated a sense of personal motivation, reflected in the goals of one member of support staff at S1, “to stabilize the psycho-emotional state and find additional motivation for victory”. Thus, The Games were an opportunity for participants to feel reinvigorated following their experiences with the conflict and determined regarding what waited for them on their return home.

Secondly, and of even greater importance, were motivations associated with raising awareness and sharing information with other nations about the experiences of Ukraine.

To present Ukraine not only on the sports field, but also in the information field, where we need significant efforts to achieve certain goals on the international front, to attract new support, to attract friends, to convey information that the war has not stopped, it continues, and the price for it is extremely high. It’s thousands of innocent human lives of Ukrainians (Active-duty personnel, S2)

This event was likely the only opportunity that many would have to speak to others about the conflict on an international platform and to “cover events in Ukraine, drawing attention to them” (Serving veteran, S1).

Now, during the war in our country, it is important that Ukraine is seen and heard on all international platforms, I want to be useful for my country and my people (Serving veteran, S2).

Similar politicised functions of sports have been identified elsewhere, including among activists of the Israeli–Palestinian conflict (46), and college athlete activists during the Black Lives Matter Movement (47). In these contexts, sports activities and events become a vehicle for education, raising awareness, collation building, and conversation, both one-to-one with individuals, and at an institutional level (46, 47).

Third, beyond the sharing of information, some also considered The Games a means of generating good news to take back to comrades, with a successful Games offering a morale-boost for those fighting in Ukraine. One veteran at S1 reflected on this as the main reason for taking part, “To motivate those who will return from war”.

… the desire to compete, win and go home with positive news to re-join the fight (Support staff, S2)

…participation of the Ukrainian team will accelerate the victory over the aggressor (Support staff, active-duty personnel, S2)

Finally, as the above comment suggests, there was a view from some that sporting success was a demonstration of national strength. In S1, one serving veteran commented that their reason for participating in The Games had been to “To prove that Ukrainians are invincible”, and another veteran, “We want to show the whole world how strong and indomitable we are”. As in past conflicts (48), sporting prowess was considered analogous with the power and resilience of the nation during the conflict and a means of demonstrating strength to both Ukraine and the world (49). Thus, whilst some participants felt guilt for leaving military duties, loved ones and comrades at home, they also perceived participation in The Games as a means of contributing to the war effort.

Sports’ ability to foster feelings of national pride and identity may be particularly important to those impacted by conflict (48, 50, 51). In this context, sports competition and team success may contribute to feelings of national unity; as Dolan and Connolly (52) describe, it offers a way of ’strengthening solidarity in the face of real or imagined antagonistic outsiders’ (p. 190). During times of conflict, then, sports participation and attendance at sporting events may not only be a route to improving physical fitness, individual well-being and morale for those participating, but also increasing morale and well-being across the nation.

### The sporting experience

When asked at S1 what their motivations for participating in The Games were, several participants reflected on sports and performance-related goals.

Perform well at the competitions and represent my country (Active-duty personnel, S1)

I want to reach if not at the peak of my physical and professional training, then at least a very good level, thereby winning medals for Ukraine, and raising the Ukrainian flag on the pedestal (Veteran, S1)

Both The Games and the training camp involved intense and high-level sports participation, with opportunities to work on sports-related skills, to compete, and to succeed in their sport. For many, success was viewed as communal, and a culmination of the team's hard work and persistence in the face of adversity.

An improvement of the results I had in the Hague [Invictus Games 2022] (Veteran, S1)

To achieve the sports result for which we prepared stubbornly and for a long time (Serving veteran, S2)

However, success was also important at an individual level. As in existing research exploring sporting motivations amongst those with disability (53, 54), the opportunity for participants to prove their capabilities to, and accomplish success for, themselves was evident.

I want to take part in competitions. The reason is to show myself, that I can really compete decently and show a good result (Active-duty personnel, S1)

[To get] good results for my national team and myself (Serving veteran, S2)

Existing literature surrounding competitive sporting participation for athletes both with and without disability has identified similar motivations, including opportunities to demonstrate talent and abilities to others (53, 55), to achieve performance-related goals and develop sporting skills (54, 56, 57) and to represent one's nation (55). Sporner et al. (12) found that for attendees at the National Veterans Wheelchair Games and Winter Sports Clinic, sports-related benefits included increased knowledge of sports equipment, and opportunities to learn sports and to be competitive.

Alongside improvements in sporting abilities and opportunities to try new sports, came a sense of achievement, as well as increased self-confidence and determination.

Added confidence in my own strength and potential (Serving veteran, S3)

Confidence and strength, gave extra determination in my plans for the future (of course, after defeating the “restless” neighbour (Active-duty personnel, S3)

The positive impact of competitive sporting participation on factors such as confidence, self-esteem, and feelings of motivation have been observed across elite and amateur athletes with and without disability (30, 53, 58), including veteran athletes with disability (13).

### The impact of the ongoing conflict on training and preparation

Despite performance-based successes, participants highlighted a number of practical implications of the war which had impeded the team's ability to prepare effectively for The Games. Logistical challenges relating to funding, resources, transport, and travel outside Ukraine were reported, as well as the difficulty of trying to train in a hostile environment. When asked about concerns prior to attending the training camp, the most common reflection from those who had undertaken active military duties was the limited training that they had been able to undertake, due to a lack of time and appropriate conditions.

The difficulties arose in the fact that since we have a war and hostilities, we do not have the opportunity to prepare for the competition (Serving veteran, S1)

I faced the fact that a large-scale war had broken out in our country, and preparations for the Warrior Games were suspended due to hostilities. Since I am a regular soldier and am currently serving in the Armed Forces of Ukraine, the training was very problematic for me (Active-duty personnel, S1)

Reflecting in S3, participants acknowledged that without the UK-based training camp, Team Ukraine would have struggled to achieve sporting success at The Games.

Of course, without training in Britain, our results would have been much worse, because almost the entire team did not have the opportunity to train in Ukraine (Active-duty personnel, S3)

Lack of preparation not only related to physical fitness and sporting technique, but also to mental preparedness for the upcoming Games.

It was very difficult to find the strength to go to training after completing the tasks… It was also very difficult to prepare mentally in the conditions of constant shelling by missiles of various classes (ground-to-ground, air-to-ground). This sometimes prevented full-fledged training and full commitment to the process (Active-duty personnel, S1).

Research highlights that for those competing in sports, psychological preparedness is a key factor in feeling ready for events and achieving peak performance (53). Any individual impacted and/or actively involved in an ongoing conflict is likely to experience limitations on their training in the run up to events, with implications for their mental readiness, particularly given the emotional toll that was associated with conflict in the current study. Those involved in the planning of competitive sports training of those involved in, or recently impacted by, conflict may need to take into even greater consideration the psychological aspects of preparing for competitions. They may benefit from integrating skills training relating to vital aspects of mental preparation in sports into competition preparation. This could include psychological self-regulation, focus, confidence and self-esteem, and strategy (53, 59).

### Relationships

Central to the enjoyment and success of participants were their experiences as a member of a well-functioning team. Participants reflected on the role of fellow members of Team Ukraine and their “Team unity” (Support staff, S2) as a source of both motivation and emotional support during training and The Games.

It seems to me that we need each other in preparation for these competitions. Because this is a colossal support and motivation (Family member, S1)

A big role in these competitions and such high results in the number of 93 medals was played by the motivation and spirit of our team (Active-duty personnel, S3)

Indeed, when asked about any positive or negative aspects of having been involved in The Games, one participant expressed worry about the end of the competition, and the difficulty of parting ways with the team.

There is some concern, because during this time our team has become like a big family, and therefore it is sad to part (Active-duty personnel, S3)

Research surrounding disabled veteran sports has commonly highlighted the role of sport and physical activity in improving social well-being, and providing opportunities for team mates to work together, offer practical and emotional support to each other, and focus on the achievements of others (1).

### International community and the spirit of The Games

Relationships outside the team were also important, and participants reflected on the sense of international community during training and competition, including their experiences of spending time with veterans from other nations. When asked about reasons for taking part (S1) and what they were most looking forward to (S2) about the upcoming games, making new friends and acquaintances from different nations was important to several participants.

…See friends from different countries who I have met before, meet new veterans from other countries (Active-duty veteran, S1)

Research shows how veteran-specific sporting events offer benefits associated with spending time with other veterans and reconnecting with military identity and values (2, 13). Roberts et al. (13) found that for Invictus Games participants, creating connections with other participants encouraged a sense of belonging at the event. Caddick and Smith (1) describe “camaraderie” in relation to the positive social interactions and emotional ties developed between veterans, and in particular, combat veterans. This, they suggest, reflects shared traumas and military experiences which enable veterans to understand and communicate with each other easily. For those with disability, this may be particularly important in developing relationships with those who understand the nature of their injury or impairment.

There was also reference to the “Spirit” of The Games, which reflected the welcome and support received by Team Ukraine from their hosts in the UK and other competing nations.

Their [British people] support makes you grit your teeth and improve your performance and prepare for competitions with more intensity and determination! (Active-duty personnel, S1)

Your [UK] support gives us wings (Serving veteran, S2)

Very impressed by the warmth of the reception of both the British and the Americans. It was like a family, one generally feels at home (Active-duty personnel, S3)

Within this environment of sporting community, supporting team members was valued more highly than personal achievements, as one member of support staff recalled:

The most important thing is to have a team and be able to count on each other. I will tell you about one of the cases that had a great impact on me. On one of the days, we had a cycling award ceremony and a wheelchair basketball competition. Half of our team chose to go to the awards ceremony to receive their medals. When I saw a member of the [team details redacted] at the competition and asked why he was not at the awards ceremony, but here, he replied: “Because the medal is already mine and that will not change. I can get it later. I have to be here, the team is counting on me.” (Support staff, S3)

Parallels can be seen here with Cree and Caddick's (60) description of the “Invictus spirit” as an “idealized military subjectivity, built on notions of brotherhood and respect” (p. 270). The Games appeared to foster a similar environment built on support, teamwork, and community. Thus, contrary to the preconceptions of one participant, who assumed a focus on sporting rivalry, The Games offered a shared communal sporting experience, built on mutual support and respect.

I expected that the Warrior Games compared to the Invictus Games is a more competitive and sporting event. Instead, this myth was completely shattered when two participants from different teams ran hand in hand or when the Canadian team came to us in the stands to support (Support staff, S3)

Despite the fact that the competition is held far from home, our native country, we were supported by absolutely all present teams, fans, representatives of the diaspora (Active-duty personnel, S3)

For one member of support staff, this communal experience had implications for the development of valuable international working relationships.

I worked quite closely with the medics, especially those… who make up the Air Force… A very strong relationship was established, we agreed to communicate in the future. I… seriously felt the support and desire to help (Support staff, S3)

Beyond the sporting opportunities available to participants, then, The Games and preceding training camp offered valuable opportunities to make and develop friendships. Perhaps most importantly, connections made with representatives from other nations, and the support received from competitors and audience members from these nations, was viewed by participants as evidence of international support for Ukraine and its people during the ongoing conflict. As one family member reflected, the experience demonstrated, “how other countries of the world are proud of Ukrainians and support us” (S3).

Friendship and support of Ukraine by ordinary people (Active-duty personnel, S3)

Findings suggest that the social opportunities provided by international sporting events may be of even greater importance to nations during times of conflict; not only a means of sharing information, but as an opportunity to integrate into an international community, and to foster positive personal and working relationships.

### Veterans in sport

As early as S1, participants were looking ahead to their sporting plans beyond The Games, with their experience of training and competing acting as motivation for future sports participation.

After these competitions, I plan to join the Paralympic team of Ukraine and professionally engage in [sport redacted] and become a Paralympic champion in the future (Serving veteran, S1)

It was evident that role models encountered at The Games had greatly influenced participants’ thinking about themselves and the future; the participation and success of other veterans helped some to view themselves and their abilities more positively and remove the limitations that they had imposed on themselves as a person with disability.

If they can, I can too! Upon my return, I try to challenge myself—Both on the battlefield and in sports (Support staff member, S3)

I considered myself old and thought that all my achievements were already in the past, but at the competitions I met [people] who achieved incredible success, they motivate me. Now I know for sure that we still have many achievements in future (Serving veteran, S3)

Conversely, participants were also motivated by the hope that taking part would benefit other veterans. The Games were viewed as an opportunity to both encourage veterans to participate in sports, and contribute to developing veteran sports in the future.

I can say that, for me, motivation is the fact that you can use your example to motivate other brothers (Veteran, S2)

This experience is priceless, not everyone gets the same opportunity as me to represent their country at competitions of this level, I will use this experience to engage even more wounded soldiers to sports (Veteran, S3)

To improve my state of health, as well as to involve as many veterans as possible in sports (Serving veteran, S1)

Similar motivations were identified by Roberts et al. (13) in Invictus competitors, who reflected on their desire to be a role model for others, particularly friends and family, in order to demonstrate to others their ability to succeed as a person with disability. Likewise, events such as the Paralympics have also been found to empower athletes with a disability, by providing them with sporting and lifestyle role models, just as in the current study (61).

### A route to rehabilitation, reintegration and changing perceptions of veterans

There were also members of support staff who were motivated by a goal to support veteran athletes in sporting endeavours as part of their wider rehabilitative journey. They reflected on the opportunities that The Games had provided for learning in this area, and their hopes for “further development of adaptive sports” (S3) and to “implement future projects for veterans considering the experience of these Games” (S3). One family member reflected similarly on their hopes of the continued rehabilitative impact of The Games on participating veterans.

I count on the fact that this tournament will also continue to be a support for disabled soldiers, and preparation for it will be an efficient rehabilitation for them (Family member, S2)

Given the ongoing conflict, the development of rehabilitative provision for Ukraine armed forces is of obvious importance; the physical, mental, and social benefits acknowledged by participants in relation to sporting and competitive participation might all help to inform the design and delivery of rehabilitative sports programmes and events in the future. This may also have wider implications for the reintegration of Ukrainian veterans into civilian life following service. As one member of support staff commented:

I hope this tournament will help highlight the issue and importance of rehabilitation for the military. And that this initiates further activities, the opportunity to reintegrate veterans (Support staff, S2)

Indeed, one veteran felt that The Games were an opportunity “to promote veteran sports in order to improve the image of a veteran” (S1), his main reason for taking part. The representation of veterans with disability in sports may be a valuable means of reframing beliefs relating to their experiences and abilities, with important implications for their journey beyond their military service. Literature surrounding veteran sporting events such as the Invictus Games suggests that they hold power and influence over societal beliefs regarding modern veterans, not least due to media coverage of these events which champions the resources that veterans draw on to overcome disability and manage rehabilitation (62).

## Summary and implications

Findings from the current study provide insight into the motivations, benefits and challenges associated with Team Ukraine's preparation for, and participation in, the 2022 Warrior Games. This team had been impacted by the ongoing conflict in Ukraine, many as serving personnel or veterans returning to active military duties. This included the death of loved ones and comrades, family displacement, and concerns about friends and relatives who remained in Ukraine.

Several benefits of the camp and participation in The Games were identified. As in previous research surrounding veteran sports, the opportunities to improve sporting abilities and to compete were important to many. However, against the backdrop of the ongoing conflict, training for the upcoming Games had been impeded by practical barriers such as limited resources and time, and the lack of safe and appropriate training environments. Findings highlight the challenges of delivering sports activities and training in this context and suggest that for those who remain living and working in a nation at war, these barriers may well limit participation and the benefits of sporting activities. Participants also reflected on their inability to prepare mentally for competition in this environment, with impacts on their confidence in the run up to The Games and, potentially, their success during competition. Such findings have important implications for the design and delivery of sports training and activities for participants impacted by conflict, not least the value of international support in the provision of a safe and resourced training environment, and the value that psychological skills training may have for competitive athletes during conflict.

The opportunity to be a role model to others was valued by participants as a means of promoting veteran sports and encouraging participation from other veterans. Likewise, participants observed the positive impact of seeing other veterans succeed, demonstrating how limitations they had imposed on themselves due to their disability might be overcome. The Games, then, altered their self-view as a person with disability, encouraging participants to set themselves new sporting challenges for the future. Sporting events may be a useful platform through which to encourage sports participation amongst not only veterans, but also others living with disability, further highlighting the value of these activities in achieving positive physical and psychological outcomes.

Being part of a close-knit team, as well as developing relationships with those from other nations, were acknowledged as both motivating factors for participants, and benefits of taking part. Whilst research surrounding veteran sports has highlighted the importance of social connection and friendship as a motivator for, and benefit of, participation, these outcomes may have been of even greater importance to members of Team Ukraine at this time. During the ongoing conflict, the ability to make connections with those outside Ukraine was considered extremely important, and signified to participants a sense of international support for Ukraine. For those working in supporting roles, the opportunities to develop strong working relationships may have important implications for the development of veteran sports in Ukraine, demonstrating the value of international knowledge sharing as a means to develop best practise in the field of veteran sports. It may be valuable for organisers of similar future competitions to promote knowledge sharing through formal means at events, such as seminars and presentations, and following events, via online forums or online events.

It should be noted that for Team Ukraine, the bond which had developed was considered both a significant benefit and motivating factor for participants, and a potential cause for concern. With The Games coming to an end, there were reflections on what the negative impact of leaving this close-knit team might be for participants. In contrast to Roberts et al. (13), who identified “post-games blues” due to concerns regarding a loss of motivation and lack of future goals, participants in the current study were more focused on the negative impacts of leaving the secure and supportive social environment of Team Ukraine. It is likely that such concerns were heightened due to the inevitable return of participants to the realities of living and working in a conflict zone, and faced with imminent danger and threat to both their own lives, and those of comrades and loved ones. Roberts et al. (13) suggest that interventions aimed at preparing athletes for “post-games blues” may be valuable. For participants in the current study, concerns regarding the end of The Games could be addressed by ensuring that team contact is maintained; there may be practical limitations on this due to the ongoing conflict and return to military duties, but the use of a private channel on a secure messaging app could provide one solution to keep team members connected. This channel would provide opportunities for participants to share their experiences and stories from The Games, to motivate each other for future sporting endeavours, to organise catch-up calls, and plan towards a team reunion or other events. Appropriate post-Games support may play an important role in ensuring the best well-being outcomes for participants, particularly for those whose well-being may be threatened by the negative impacts of an ongoing conflict.

The rehabilitative role of sports participation was acknowledged across the sample. This included benefits to sporting ability, strength and fitness, but also emotional rehabilitation. These benefits were felt amongst both those involved in front line duties and family members impacted by the death of loved ones during the war. The continued work of The Ministry of Veterans Affairs of Ukraine and its National Strategy for physical and sports rehabilitation of veterans and families demonstrates the growing acknowledgement of the value of sports to the lives of those impacted by conflict (29, 30). Given the ongoing nature of the Russo-Ukrainian conflict and the impacts on both those actively involved, and witness to, the war, insight into how sports and exercise may impact on psychological, physical, social, and rehabilitative outcomes may be valuable to the development of practice and policy in the wake of the conflict. Although, consideration of military, veteran and civilian sporting experiences, including rehabilitation, training, and competitions, within Ukraine itself is also needed to evaluate the longitudinal impacts of policies and programmes during and following the war. Still, it is hoped that the current study provides useful insight into the experiences of this unique group during the current conflict, helping to inform the nation's engagement in future veteran sporting events.

Consideration of the sporting experiences of those living in other countries impacted by war could also help to identify best practise and inform future sporting programmes and events globally. In particular, the use of qualitative interview techniques could provide detailed insight to the sporting experiences of these groups, including any benefits, barriers, or disadvantages associated with participation. Such research could also shed light on possible cultural differences in the motivations and experiences of competing nations at international veteran and non-veteran sporting events, the impact of socio-political factors such as conflict on these experiences, and how individual and collective goals are achieved within the context of competitive sports. Exploration of the role of recreational sports activities, competitions, and sports-based rehabilitation in the lives of those impacted by other humanitarian crises may also be valuable in understanding the extent to which benefits and challenges are experienced by both participants and providers in these contexts.

Finally, perhaps one of the most common sources of motivation for taking part in The Games amongst participants was the ongoing conflict in Ukraine. This manifested in several ways, including as a source of motivation and determination for victory, a means of generating positive news to take home to comrades, and as a way of demonstrating national strength through sporting success. However, it was the opportunity to raise awareness of the ongoing conflict and the experiences of the Ukrainian people within the international community that was considered of greatest importance. Beyond simply participating in sports, participants sought opportunities to engage politically with the international sporting community, with the sharing of information considered a contribution to the ongoing war effort. As Meeuwsen and Kreft (63) write, Ukrainian athletes who have chosen to appear in sport competitions during the conflict have stated that they do so because “their presence encourages Ukrainian soldiers and Ukrainian people to resist” (p. 349). This finding contributes contemporary insight to the field of sport and politics, highlighting that for the athletes themselves, sporting events may hold important political functions, particularly during times of conflict. This confirms that the divide made between sports and politics by international sporting authorities and committees such as The International Olympic Committee, may be an impossible principle to uphold (63, 64). Future research which seeks to bring the voices of all those involved in sporting events, including athletes, audience members, volunteers, and organisers, to discussions of politics and sports may shed further light on the significance of these events during times of both conflict and peace. If the politicisation of sports is unavoidable, it is essential to understand what the implications of this are for the individuals and nations involved, and how this might help or hinder political objectives.

## Limitations

The key limitation of the current research was the specific context in which the work was developed and run. The work was to be delivered within a short time period, there was a need to survey participants as soon as possible following their arrival in the UK, surveys had to be completed around busy training schedules, and following The Games, it was likely that some participants would be unreachable due to their return to military duties. With a short notice period for Team Ukraine's arrival, it was not feasibly possible to pilot qualitative questions with the target sample. However, the questions were translated and checked for sense by a member of support staff working with Team Ukraine to ensure suitability for use with the team, including appropriate language use. Furthermore, Team Ukraine had direct contact with one member of the research team during their stay, through whom any issues or concerns regarding the surveys could be raised. A further limitation is the translation of responses from Ukrainian to English. Professional translations of responses were sense-checked by the support staff member from Team Ukraine but even so, this additional step prior to the analysis process may have impacted on interpretation of the data. Thus, results should be viewed within this unique context, offering an indication of the key experiences and motivations for participation amongst Team Ukraine. It is likely that greater insight would have been available with greater time to prepare and validate questions, and the use of qualitative interviewing techniques with a Ukrainian speaker to allow for more detailed exploration of experiences.

## Conclusion

This article provides novel insight into the experiences of a sports team preparing for, and competing at, an international sporting event for veterans with disabilities during an ongoing conflict in their home country. Results demonstrate the range of motivational factors influencing decisions to participate, including the perception of The Games as an international platform on which to raise awareness of the conflict. Benefits to physical and emotional health, and the importance of social aspects of both the training camp and The Games, were highlighted. Future research is needed to explore, in greater detail, the sports participation of both military and civilian populations from Ukraine, and other countries, during and following conflict. This topic remains underexplored. Such research should include experiences associated with recreational, rehabilitative, and competitive sporting activities. Findings have implications for understanding the role that sports plays during periods of socio-political uncertainty and in the lives of those impacted by social and natural disasters, as well as helping to inform practise and policy for sports activities and events run in these contexts in the future.

## Data Availability

The datasets presented in this study can be found in online repositories. The names of the repository/repositories and accession number(s) can be found below: FigShare, 10.6084/m9.figshare.27636543.

## Appendix. Survey questions

### Survey 1

(1) What is the main reason you decided to take part in this year's Warrior Games?
(2) Could you describe any concerns you had about taking part in this year's Warrior Games?
(3) Thinking about the time before your arrival in the UK, what were some of the challenges you experienced in relation to preparing for the Warrior Games?
(4) Thinking about the upcoming weeks, what are you hoping to gain from your time preparing here in the UK?
(5) And finally, is there anything else you would like to tell us about your preparations for the Warrior Games so far?

### Survey 2

(1) Could you briefly describe how you feel your preparations for the Warrior Games have gone?
(2) Looking back on your preparations for the Warrior Games, what do you feel have been the benefits of the time you have spent here in the UK?
(3) What has kept you motivated during your preparations for the Warrior Games?
(4) Could you describe any challenges you have experienced during your preparations for the Warrior Games in the UK?
(5) What are you most looking forward to about the upcoming Warrior Games?
(6) Finally, is there anything else you would like to tell us about your preparations for the games or your experience in the UK over the last few weeks?

### Survey 3

(1) Could you reflect on anything you have learned, or any skills you have developed, during your time preparing for, and attending, the Warrior Games?
(2) Do you think that any of the skills you have developed, or experiences you have had, will help you in your life back in Ukraine? If yes, could you tell us how.
(3) Could you tell us about any experiences that stood out as particularly significant or memorable during your time preparing for, and attending the Warrior Games?
(4) Could you describe your experience of being a member of team Ukraine and representing Ukraine at the Warrior Games?
(5) What impact, if any, do you feel competing at the Warrior Games has had on you as a person, including how you feel about yourself, your life overall, and the future?
(6) Could you describe any positive or negative impacts of your involvement in the Warrior Games on your overall health, well-being, or morale?
